# Transcriptional profiling of uterine leiomyoma rats treated by a traditional herb pair, *Curcumae rhizoma* and *Sparganii rhizoma*

**DOI:** 10.1590/1414-431X20198132

**Published:** 2019-05-27

**Authors:** Cheng Hao Yu, Jin Shuang Zhao, Hui Zhao, Teng Peng, Dong Cheng Shen, Qiu Xia Xu, Yao Li, R. Clinton Webb, Mong Heng Wang, Xing Ming Shi, Cheng Peng, Wei Jun Ding

**Affiliations:** 1 Chengdu University of Traditional Chinese Medicine, Chengdu, Sichuan, China; 2 Medical College of Georgia, Augusta University, Augusta, GA, USA; 3 The First Affiliated Hospital of Henan University of Traditional Chinese Medicine, Zhengzhou, Henan, China; 4 The Community Health Service Center of Xi'an Road, Chengdu, Sichuan, China; 5 University of Chinese Academy of Sciences Shenzhen Hospital, Shenzhen, Guangdong, China

**Keywords:** Uterine leiomyoma, Transcriptional profiling, Extracellular matrix, Curcumae rhizoma, Sparganii rhizoma

## Abstract

The aim of this study was to elucidate the concise effects of a traditional herb pair, *Curcumae rhizoma-Sparganii rhizoma* (CRSR), on uterine leiomyoma (UL) by analyzing transcriptional profiling. The UL rat model was made by intramuscular injection of progesterone and gavage administration of diethylstilbestrol. From 11 weeks of the establishment of the model, rats of the UL+CRSR group were gavaged daily with CRSR (6.67 g/kg). The serum concentrations of progesterone (P) and estradiol (E_2_) were determined by radioimmunoassay, the uterine index was measured by caliper measurement, and the pathological status was observed by hematoxylin and eosin stain. Gene expression profiling was checked by NimbleGen Rat Gene Expression Microarrays. The results indicated that the uterine mass of UL+CRSR rats was significantly shrunk and serum P and E_2_ levels significantly reduced compared to UL animals and nearly to the level of normal rats. Results of microarrays displayed the extensive inhibition of CRSR upon the expression of proliferation and deposition of extracellular matrix (ECM)-related genes, and significantly regulated a wide range of metabolism disorders. Furthermore, CRSR extensively regulated key pathways of the UL process, such as MAPK, PPAR, Notch, and TGF-β/Smad. Regulation of the crucial pathways for the UL process and ECM metabolism may be the underlying mechanisms of CRSR treatment. Further studies will provide clear clues for effectively treating UL with CRSR.

## Introduction

Uterine leiomyoma (UL, also called uterine fibroid) is a common, non-malignant, smooth muscle tumor, affecting 30 to 70% of females during their reproductive periods (1), having a tremendous medical and economic impact worldwide (2), costing approximately US$ 5.9–34.4 billion annually (3). UL causes abnormal uterine bleeding, pelvic pain, bladder or bowel dysfunction, and subfertility. The proliferation of leiomyoma cells together with excessive deposition of extracellular matrix (ECM) is the crucial pathological process. These two mechanisms are tightly regulated by a complex network of interconnected pathways, including pathways of steroids, growth factors, TGF-β/Smad, Wnt/β-catenin, retinoic acid, vitamin D, and PPARγ (1,2,4). Interestingly, aberrant signal transduction pathways in UL patients are not only intracellular, but also extracellular and intercellular pathways. Furthermore, partly due to the poor understanding of the underlying pathobiology, there is no effective medical treatment currently available (1,4,5). Major therapy strategies are based on the inhibition of estrogen or progesterone, but UL tends to regrow once treatment is stopped. Therefore, understanding aberrations of these signaling pathways and their interconnections is critical for the development of satisfactory drugs.

Herbal preparations are commonly used as alternatives to surgical procedures. Yen and colleagues analyzed 35,786 diagnosed UL subjects in Taiwan, of whom 53.8% had used Chinese herbal remedies (6). Jacoby et al. (7) reviewed that complementary and alternative treatments were extensively used in ULs, including exercise (45%), diet (34%), herbs (37%), and acupuncture (16%). Participants reported significant symptom improvement and few side effects with these interventions. Both clinical observations and animal research demonstrated that Chinese herbs can reduce the leiomyoma volume and shrink the size of the uterus. In fact, only 10 to 20% of UL patients require surgery; interventions such as traditional Chinese medicine (TCM) may eliminate the need for surgery in some cases, especially if treated early (8). TCM can also reduce the consumption of conventional chemical drugs (9).

Meta-analyses showed that *Curcumae rhizoma* and *Sparganii rhizoma* were among the frequently prescribed Chinese herbs for UL treatment (7–10). *S. rhizoma* exhibits anti-angiogenesis, anti-inflammation, and anti-estrogen effects (11–14). *C. rhizoma* is widely used in the therapy of UL but absolutely prohibited for the pregnant women in clinics (15–17). It possesses anti-inflammatory and anti-tumor activities and responds to multiple regulation of signaling pathways for ECM deposition (18), cell cycle arrest (19), and UL process (20). Moreover, TCM has long established the concise and synergistic effects of *C. rhizoma* and *S. rhizoma*, and, as a consequence, a compound with both was developed for UL therapies (21). Couplet medicines in TCM concept refer to two herbs that exhibit synergistic pharmaceutical and/or detoxic activities. *C. rhizoma*-*S. rhizoma* (CRSR) is a classical herb pair in clinics of TCM, and it has been used for thousands of years. ULs belong to the concept of “Zhengjia” and “Jiju”, which are caused by “blood stasis” and “Qi stagnation” in TCM. CRSR is used for activating blood circulation and breaking the “Qi”, and treating especially gynecologic tumors in TCM clinics (22).

Although CRSR possesses synergistic effects based on TCM theories in UL therapies, the underlying mechanisms are not clear. Here, we primarily verified the CRSR effect against UL based on the approach of transcriptional profiling.

## Material and Methods

### Chemicals and reagents

The dried herb of *C. rhizoma* was purchased from Sichuan Kelun Pharmaceutical Co. Ltd (China). The crude herb was crushed into powder and sieved by a 60-mesh sieve. According to the extraction method of volatile oil recorded in the Chinese Pharmacopoeia (2010 Edition), the volatile oil of *C. rhizoma* was extracted by steam distillation. Then, the volatile oil was collected for further use.

For preparation of the total flavonoids of *S. rhizoma*, the dried *S. rhizoma* was also purchased from Sichuan Kelun Pharmaceutical Co. Ltd. The crude herb was smashed into powder and sieved by a 60-mesh sieve. Total flavones were extracted with 10× volume of sample weight with 60% alcohol for three times, and 1.5 h each time. Finally, the above filtrates were combined, vacuum-dried to constant weight, and stored at 4°C for further analysis.

Diethylstilbestrol tablets (Cat No. 20100920, 0.5 mg/tablet) were made by Hefei Jiu Lian Pharmaceutical Co., Ltd. (China). Progesterone for injection (Cat No. 120307, 10 mg/mL) was made by Zhejiang Xian Ju Pharmaceutical Co., Ltd., China. The estradiol radioimmunoassay kit (Cat No. E10J015) and progesterone radioimmunoassay kit (Cat No. E10J023) were purchased from Beijing Fu Rui Biological Engineering Co., Ltd., China. DAB color kit (Cat No. 12182A01), horseradish peroxidase-labeled streptavidin working solution (Cat No. 12122A08) and citrate buffer (pH 6.0, Cat No. ZLI-9065) were purchased from Beijing Zhong Shan Golden Bridge Biotechnology Co., Ltd., China.

### Animal model and herbal treatment

Thirty female Sprague Dawley rats, weighing 200±20 g, were obtained from the Experimental Animal Center, Chengdu University of TCM (China). After adaptive feeding for 1 week, rats were randomly divided into 3 groups: normal control (NC, n=10), UL (n=8), and CRSR therapy (UL+CRSR, n=10). All experiments were approved by the Institutional Animal Care and Use Committee of Chengdu University of TCM. Rats of both UL+CRSR and UL groups received diethylstilbestrol (0.167 mg/kg) by gavage every other day, and intramuscular injection of progesterone (1 mg/kg) once a week, for 20 weeks. Animals of the NC group were maintained in the same conditions without any treatment. From 11 weeks of model-induction, rats of the UL+CRSR group were gavaged daily with the volatile oil of *C. rhizoma* and the total flavonoids of *S. rhizoma* (6.67 g/kg), 10 times the clinical dosage for human beings (w/w), which was calculated based on the crude herbs used for the extractions. Animals were observed daily in order to record any post-treatment complication or adverse event.

### Sample collection

At the end of the 20-week experiment, animals were anesthetized with ether and the following procedures were conducted. Blood sample was collected from the femoral artery and centrifuged at 1048 *g* and 5°C for 15 min. The serum was collected and the concentrations of progesterone (P) and estradiol (E_2_) were immediately determined by radioimmunoassay kits, in accordance with the supplier's instruction. Length and root diameter of the uterine horn were measured by caliper measurement. The uterine coefficient was calculated using the formula: uterine coefficient = uterine weight (mg) / rat body weight (g) × 100%. UL tissues were collected from all rats in areas adjacent to the leiomyoma and from the endometrial, intramural, and serosal layers at a location distant from the leiomyoma within the uterus. In the normal control animals, tissue samples at the same areas were collected. Uterine tissues were divided into two parts: one was immediately preserved in liquid nitrogen and then stored at –80°C for transcriptional analysis and the other was formalin-fixed for 15–20 h, paraffin-embedded, and subjected to hematoxylin and eosin (H&E) histological observation.

### Transcriptional profiling analysis

Gene expression profiling analysis was performed using NimbleGen Rat Gene Expression Microarrays (Roche NimbleGen, USA) (135 K arrays covering 26,419 UniGenes collected from NCBI). Total RNA from each sample was quantified by a NanoDrop ND-1000 (Thermo, USA) and RNA integrity was assessed by standard denaturing agar gel electrophoresis (Biowest Agarose, Spain). Five micrograms of total RNA of each sample was utilized for labeling and array hybridization with the following steps: 1) reverse transcription by the Invitrogen Superscript ds-cDNA synthesis kit (USA); 2) ds-cDNA labeling with NimbleGen one-color DNA labeling kit (Roche NimbleGen, USA); 3) array hybridization using the NimbleGen Hybridization System followed by washing with the NimbleGen wash buffer kit; 4) array scanning using the Axon GenePix 4000B microarray scanner (Molecular Devices Corporation, USA). Scanned images were then imported into NimbleScan software (version 2.5) for grid alignment and expression data analysis. Expression data were normalized through quantile normalization and the Robust Multichip Average (RMA) algorithm included in the NimbleScan software. The Probe level (*_norm_RMA.pair) files and Gene level (*_RMA.calls) files were generated after normalization. The gene data were imported into Agilent GeneSpring GX software (USA, version 11.5.1) for further analysis. Genes that had values greater than or equal to the cut-off of 100.0 were chosen for data analysis. Microarray data could be shared by relevant experts for academic research upon request. Differentially expressed genes were identified through fold-change filtering. Pathway analysis and Gene Ontology (GO) analysis (http://geneontology.org/) were applied to determine the roles of these differently expressed genes in biological pathways or GO terms. Finally, hierarchical clustering was performed to show different gene expression profiling among samples.

Genes that were found to be significantly different in a pairwise comparison were further analyzed for canonical pathways, networks, transcription factors, and biological functions using the Ingenuity Pathway Analysis (IPA) software (Ingenuity Systems, USA). The IPA software is based on computational algorithms of the connectivity from information obtained within the IPA database. IPA analysis accounts for the type of chip used with a score assigned to rank networks according to their relevance to the gene list provided. Canonical pathways were determined by analyzing a ratio of the number of genes that map to the pathway, divided by the total number of genes in the pathway that are represented by the chip probes, and the P value was calculated by Fisher's exact test to determine the probability that the association was due to chance alone.

### Statistical analysis

The statistical software SPSS 16.0 was used to carry out all data analysis. Results of quantitative experiments are reported as means±SD. Each data point represents the average of three independent experiments. One-way analysis of variance was used for multiple group comparisons. When only two groups were compared, the Student's unpaired *t*-test was used. Results were considered statistically significant if the P value was less than 0.05.

## Results

### CRSR significantly reduced UL parameters

**Figure 1:**
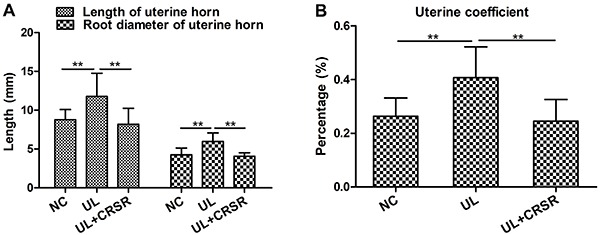
Effects of *Curcumae rhizoma-Sparganii rhizoma* (CRSR) treatment on the length of the uterine horn, the root diameter of the uterine horn (**A**), and the uterine coefficient (**B**). Data are reported as means±SD. NC: normal control, n=10; UL: uterine leiomyomas, n=8; UL+CRSR: treatment group, n=10. **P<0.01 compared with the UL model (ANOVA).

Two representative indexes of uterine size (i.e., the length of the uterine horn and the root diameter of the uterine horn) were both significantly reduced in UL+CRSR rats (P<0.01). On the other hand, there was no significant difference in the uterine size of UL+CRSR rats compared with those of the NC animals, yet some increase of the uterus was still observed after CRSR treatment (Figure 1A). The uterine coefficient in UL increased compared to NC. The uterine coefficient in UL+CRSR significantly decreased compared with UL (Figure 1B). The uterine coefficient did not differ significantly between NC and UL+CRSR (P>0.05).

### Histological changes in the uterus

**Figure 2:**
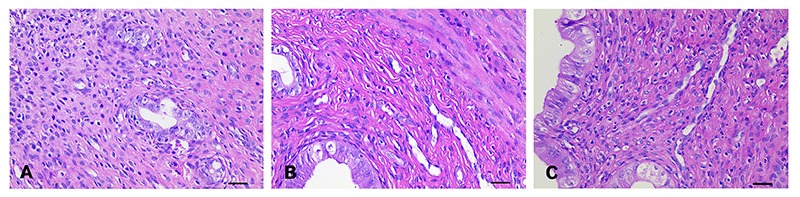
Representative micrographs obtained by hematoxylin and eosin stain. The original magnification was 400×. **A**: NC, normal control; **B**: UL, uterine leiomyomas; **C**: UL+CRSR, treatment group with *Curcumae rhizoma-Sparganii rhizoma*. Magnification bar: 30 μm.

Representative H&E micrographs from NC, UL, and UL+CRSR rats are shown in Figure 2. Fibroid sections from UL rats showed distorted, sparsely distributed nuclei with smudged chromatin pattern, and ill-defined nuclear and cytoplasm outline (Figure 2B). Major histological changes in fibroid sections from the UL+CRSR rats were mild hypertrophy and focal mild hyperplasia of smooth muscle cells, yet neatly arranged (Figure 2C). Conversely, uterine samples from NC rats showed the typical interlacing bundles of smooth muscle cells (Figure 2A). The smooth muscle cells were elongated and spindle-shaped, and had indistinct cell borders and abundant pale eosinophilic cytoplasm. The nuclei were uniform, crowded, and tightly packed with a fine granular chromatin pattern. Based on 200 representative cells from H&E micrographs derived from each group, the average ECM thickness of NC, UL, and UL+CRSR was 12.64±3.87, 21.57±5.35, and 14.24±3.49 μm, respectively. Hence, histological uterine changes confirmed the therapeutic effect of CRSR.

### CRSR markedly reduced the concentrations of serum E_2_ and P

**Figure 3:**
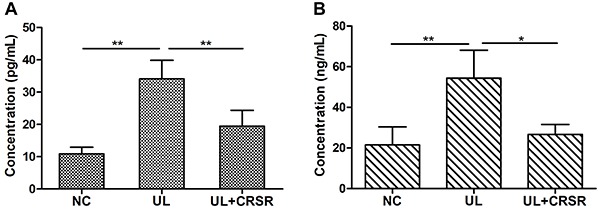
Effects of *Curcumae rhizoma-Sparganii rhizoma* (CRSR) treatment on the key hormones of uterine leiomyomas (UL): **A**, estradiol (E_2_); **B**, progesterone (P). Data are reported as means±SD. NC: normal control, n=10; UL: uterine leiomyomas, n=8; UL+CRSR: treatment group, n=10. *P<0.05, **P<0.01 (ANOVA).

High levels of serum E_2_ and P are key factors for the promotion of the UL process. We observed by standard radioimmunoassay that UL rats had significantly higher levels of serum E_2_ and P, compared with NC rats. After the CRSR treatment for 10 weeks, both hormones were restored almost to that of the NC animals (Figure 3). Therefore, these data suggest that CRSR reduced the serum hormones that otherwise can promote UL development, and subsequently led to shrinkage of ULs.

### CRSR inhibited the expression of proliferation- and ECM-related genes

ULs are characterized by an abundance of ECM, which is mainly composed of collagen and fibronectin (9). Previous reports have demonstrated that genes that encode ECM proteins were expressed abnormally in leiomyomas. Our microarray results demonstrated a large number of differently expressed genes with roles in signal transduction pathways, metabolism, cell proliferation, and ECM formation (complete data not shown). Table 1 lists the top ten down- or up-regulated genes. For instance, lipocalin 2 is associated with multiple biological processes such as pheromone transport, immune response, retinoid binding, and MMP-9 synthesis (23); aquaporin 8 is a small integral membrane protein related to ECM formation (24); the classic role of oxytocin receptor is as an inducer of uterine contractions (25). Compared with NC rats, genes of lipocalin 2 and aquaporin 8 were extremely up-regulated in UL animals, suggesting the over-expression of ECM in UL rats. These results imply that CRSR might reduce uterine size in UL animals by adjusting the ECM-associated genes and their regulation pathways (Table 1).

**Table 1:** Top ten significantly regulated genes between different groups.

	Gene symbol	Fold change	Gene symbol	Fold change
NC *vs* UL	Lcn2	69.817	Slc39a1	−9.578
	Gal	40.528	Nov	−8.501
	Aqp8	35.497	LOC682601	−7.880
	Ngfr	33.604	Lrrc17	−6.492
	Cbln1	32.587	Lrrc27	−6.108
	Rasd1	27.603	Spon2	−6.083
	Ccl7	18.854	Hmgcs2	−6.057
	Oxtr	17.999	Serpina3n	−6.049
	Gadd45g	14.546	Ctla2a	−5.660
	LOC688246	12.186	Tppp	−5.144
UL+CRSR *vs* UL	Slc39a1	15.484	Nxt1	−15.458
	Serpina3n	12.126	LOC688976	−4.128
	Hmgcs2	4.784	LOC680511	−3.998
	Cxcl6	4.650	Tpcn2	−3.987
	Il13ra2	3.105	LOC685415	−3.646
	Ccr7	3.079	RGD1560815	−3.613
	Adipoq	3.008	LOC502486	−3.608
	Wfdc10	2.995	LOC499487	−3.546
	LOC688556	2.860	LOC684897	−3.416
	Snip	2.852	LOC684285	−3.395
UL+CRSR *vs* NC	Lcn2	57.935	Nxt1	−12.546
	Ngfr	45.672	Nov	−8.293
	Aqp8	35.914	LOC682601	−7.454
	Cbln1	30.296	LOC690062	−6.763
	Rasd1	29.853	Spon2	−6.404
	Gal	19.618	Trpv6	−6.366
	Oxtr	17.296	Lrrc17	−5.744
	Gadd45g	15.439	Ctla2a	−5.743
	Gpr88	11.130	Cpm	−5.725
	Ccl7	11.077	LOC685369	−5.383

The positive and negative numbers indicate up- and down-regulated genes, respectively. NC: normal control; UL: uterine leiomyomas; UL+CRSR: treatment group with a traditional herb pair, *Curcumae rhizoma* and *Sparganii rhizoma*.

GO analysis more clearly reflected the primary patterns of the differently expressed genes between NC, UL, and UL+CRSR animals (Table 2). Results of GO biological process classification displayed that the down-regulated response to stimulus and up-regulated metabolic process were the most distinguished features between UL and NC rats. The metabolic process, biosynthesis process, response to stimulus, and developmental process were quite different between UL+CRSR and UL animals. As for UL+CRSR and NC groups, metabolic process, biological regulation, regulation of cellular and biological process, multi-cellular organism development, and cellular process exhibited differences. These results indicated that in biological process, regulation of metabolism and development, response to stimulus, and adjustment of biosynthesis process were majorly responsive for the pathological process of UL. Such conditions were also related to the pharmacological effects of CRSR.

**Table 2:** Gene ontology (GO) analysis of the differently expressed genes.

	UL *vs* NC	UL+CRSR *vs* UL	UL+CRSR *vs* NC
Biological Process			
	Response to stimulus	Metabolic process	Metabolic process
	Multicellular organism process	Biosynthetic process	Multicellular organism development
	Primary metabolic process	Regulation of biological process	Cellular component organization or biogenesis
	Metabolic process	Response to stimulus	Location
	Anatomical structure development	Developmental process	Anatomic structure development
	Multicellular organism development	Multicellular organismprocess	Regulation of biological process
	Cellular process	Protein metabolic process	Biological regulation
	Localization		Cellular process
	Regulation of biological process		Regulation of cellular process
Cellular component			
	Extracellular region	Cell	Organelle
	Extracellular matrix	Intracellular	Extracellular region
	Neuron projection	organelle	Extracellular matrix
	Axon	Intracellular organelle	Ribosome
	Perinuclear region	Cytoplasm	Ribonucleoprotein
	Intermediate filament	Extracellular region	Intermediate filament
	Organelle	Plasma membrane	Keratin filament
	Intracellular	Extracellular matrix	Cytoplasm
	Cytoplasm	Cell periphery	Intracellular
Molecular Function			
	Binding	Protein binding	Binding
	Protein binding	Structure of ribosome	Receptor binding
	Catalytic activity	Structural molecular activity	Structural molecular activity
	Metal ion binding	Microtube binding	DNA binding
	Small molecular bind	Glucose binding	Small molecular bind
	Nucleotide binding	Enzyme binding	Structure of ribosome
	Receptor binding	Magnesium ion binding	Metal ion binding
	Transferase activity	Apolipoprotein binding	Protein binding
	Cytokine activity	Peptidase regulator activity	Cytokine activity
	Hormone activity	Neurotransmitter receptor	Catalytic activity

NC: normal control; UL: uterine leiomyomas; UL+CRSR: treatment group conducted with a traditional herb pair, *Curcumae rhizoma* and *Sparganii rhizoma*.

Results of GO cellular component classification demonstrated that the down-regulated genes were majorly involved in the extra-cellular region or matrix, and up-regulated intracellular components were the most distinguished features between UL and NC animals. This indicates the abnormal metabolism of critical components was involved in the UL process. Interestingly, these abnormal metabolisms were substantially reversed in the UL+CRSR animals. Comparing UL+CRSR with NC rats, any of the extra- and intra-cellular components were adjusted, and constitution of ECM, such as keratin filaments, intermediate filament, and ribonucleoprotein, was also intensively regulated (Table 2).

Results of the GO molecular function classification showed that diverse binding functions (i.e., protein, metal ion, small molecular, nucleotide, and receptor binding) and various metabolic or immunological activities were among the differences between NC and UL animals. Comparing UL+CRSR with NC rats, adjustment of various binding functions were found, suggesting the recovered activities of diverse binding functions were related to the therapy effects of CRSR against UL process. Comparing UL+CRSR with UL rats, similar results were observed for a series of binding functions and catalytic and cytokine activities (Table 2).

### CRSR extensively regulated the key signal pathways of the UL process

The top five significant canonical pathways identified by IPA are displayed at Table 3. Nine of the down-regulated pathways were identified in the UL rats, compared to NC rats (Supplementary Table S1). These are metabolism pathways, i.e., metabolism of cofactors, vitamins, xenobiotics, amino acids, and lipids. On the other hand, 28 up-regulated pathways were identified from the uterus tissues of UL rats (Supplementary Table S2), which are mainly involved in infectious diseases, nucleotide and amino acid metabolisms, signal transduction, ECM metabolism, and regulation of the endocrine-immune-nervous web. This indicates that the UL process is involved with abnormal metabolism, excessive ECM synthesis, and disturbed signal transduction and immune function (26).

**Table 3:** Top five differentially expressed pathways identified by Ingenuity Pathway Analysis.

	Down-regulated pathway			Up-regulated pathway		
	Pathway Name	P value	Enrichment Score	Pathway Name	P value	Enrichment Score
NC *vs* UL	Pantothenate and CoA biosynthesis	0.001	2.964	Hedgehog signaling pathway	0.049	1.314
	Arachidonic acid metabolism	0.004	2.364	Purine metabolism	0.045	1.350
	Drug metabolism - cytochrome P450	0.005	2.298	Seleno-compound metabolism	0.043	1.363
	Glutathione metabolism	0.006	2.243	RNA degradation	0.041	1.385
	Metabolism of xenobiotics by cytochrome P450	0.006	2.213	Cysteine and methionine metabolism	0.033	1.486
UL+CRSR *vs* UL	Influenza A	0.001	2.884	PPAR signaling pathway	0.046	1.335
	Graft *vs* host disease	0.014	1.865	Chemokine signaling pathway	0.043	1.365
	Allograft rejection	0.017	1.778	Adipocytokine signaling pathway	0.004	2.453
	Type I diabetes mellitus	0.018	1.755	Pantothenate and CoA biosynthesis	0.002	2.660
	Gastric acid secretion	0.018	1.733			
UL+CRSR *vs* NC	Arachidonic acid metabolism	0.001	3.066	Purine metabolism	0.048	1.315
	Antigen processing and presentation	0.002	2.757	RNA transport	0.042	1.381
	Drug metabolism - cytochrome P450	0.006	2.220	Seleno-compound metabolism	0.027	1.575
	Cell adhesion molecules	0.006	2.208	TGF-beta signaling pathway	0.024	1.613
	Serotonergic synapse	0.009	2.032	Pyrimidine metabolism	0.023	1.643

NC: normal control; UL: uterine leiomyomas; UL+CRSR: treatment group conducted with an herb pair, *Curcumae rhizoma* and *Sparganii rhizoma.*

Fewer differently expressed pathways were detected between UL+CRSR and UL rats (Table 3 and Supplementary Table S3). The down-regulated pathways belonged to diseases (metabolic, digestive, immunological, or infectious diseases), and attenuated functions of antigen processing and presentation. Four up-regulated pathways were involved in pantothenate and CoA biosynthesis, PPAR, adipocytokine, and chemokine signaling. Results indicated that only limited pathways were recovered to normal status.

There were sixteen down-regulated pathways between UL+CRSR and NC rats (Supplementary Table S4). These pathways are related to metabolism (lipids, xenobiotics, and amino acids), immune system and metabolic diseases, ECM formation, and systemic regulation. We also identified 25 up-regulated pathways in UL+CRSR animals (Supplementary Table S5). These pathways are mainly related to diseases (metabolic or infectious diseases), nucleotide and amino acids metabolism, signal transductions (such as MAPK, Notch, and TGF-beta pathways), ECM metabolism, and regulation of the endocrine-immune-nervous system. These results suggested that there were still notable differences between UL+CRSR and NC rats, particularly in ECM metabolism, immune functions, and signal transduction pathways involved in fibroid formation.

Taken together, our results demonstrated that CRSR treatment can extensively and profoundly modify the signaling pathways involved in UL development and remedy processes. These include at least three approaches: 1) suppressing ECM metabolism directly; 2) regulating key signaling of UL process, such as MAPK, PPAR, Notch, and TGF-beta signaling pathways; 3) tuning signaling pathways of global metabolism and immunity that indirectly regulate the development of UL process.

## Discussion

UL is a complex disease that exerts a huge burden on healthcare resources worldwide. The management of UL is an enormous challenge given the high incidence and the lack of an effective and safe nonsurgical treatment (2,4,5,6). Although hysterectomy remains the main option for the treatment of UL, the surgical approach is not always a favorable choice, particularly in women who desire to preserve their fertility. Therefore, the development of an effective and nonsurgical therapeutic strategy is a critical need in female health care.

Chinese herbs have been extensively used in oriental countries, showing advantages in treating gynecologic disorders (6–11). Meta-analyses show that CRSR was frequently used for effective therapy of UL (12–17). High concentrations of Grailsine-Al-glycoside, the bioactive component of *S. rhizoma*, strongly suppressed cell proliferation in a dose-dependent fashion in A549, MCF-7, HepG2, and HeLa cells (13). Sparstolonin B, a bioactive compound isolated from *S. rhizoma*, inhibited endothelial cell tube formation and cell migration in part by down-regulating CCNE2 and CDC6, halting progression through the G1/S checkpoint (14). Sparstolonin B achieved anti-inflammation effects by the attenuation of LPS-induced phosphorylation of signaling molecules Erk1/2 and Akt (15). On the other hand, *C. rhizoma* is also widely used in the therapy of ULs (16). The essential oil of *C. rhizoma* presented anti-angiogenic activity *in vitro* and *in vivo*, resulting in the suppression of tumor growth and metastasis, by inhibiting the phosphorylation of ERK1/2 and AKT/NF-κB signaling pathways, and enhancing the phosphorylation of JNK1/2 and p38 (16–18). Petroleum ether extracts of *C. rhizoma* produce a significant G0/G1 cell cycle arrest at the concentration of 300 μg/mL (19). Curcumenol, a sesquiterpene isolated from *C. rhizoma*, possess anti-inflammatory and anti-tumor activities by inhibiting Akt-dependent NF-κB and p38-MAPK signaling (20). The chloroform-fraction of *C. rhizoma* significantly inhibited leiomyoma cell proliferation (21). However, the synergistic effects of *C. rhizoma* and *S. rhizoma* against UL growth have not been studied. Therefore, we explored the anti-fibroid effects of CRSR through a novel approach, transcriptional profiling.

The process of UL development is apparently regulated by the serum levels of sex hormones. Both estrogen and progesterone play crucial roles in UL growth (4,5,16). We observed significantly reduced serum concentrations of both estrogen and progesterone in CRSR-treated rats, indicating the pharmaceutical implication of CRSR (12–15). Additionally, CRSR therapy can markedly reduce uterine size represented by the length of the under point of uterine horn, the root diameter of uterine horn points, and uterine coefficient, and substantially recover the pathological conditions. These results indicated that the therapy effects of CRSR achieved the same levels as certain famous TCM formula, such as Gui-zhi Fu-Ling decoction (11), yet fewer components were recruited. Furthermore, histological observation reconfirmed that CRSR can effectively attenuate the process of ECM deposition and UL growth. Results of both pathology and endocrine assays indicated that CRSR can inhibit UL growth.

To further understand the potential mechanisms of UL shrinkage induced by CRSR, we analyzed the transcriptome features by cDNA microarrays. ULs are characterized by the excess synthesis and abundant accumulation of ECM proteins (1,2,4,5,27). Our microarray results demonstrated a large number of abnormally expressed genes that were mainly involved in cell proliferation and ECM formation, metabolism, and signal transduction pathways, suggesting that CRSR might reduce the UL process by intricately tuning the ECM-associated genes and/or their regulation pathways. Furthermore, GO analysis of these abnormally expressed genes clarified the regulation patterns among NC, UL, and UL+CRSR animals. Results of GO biological process demonstrated that global adjustment of metabolic, biosynthetic, and developmental processes, promotion of the response to stimulus, and inhibition of the ECM formation were the key points of CRSR treatment. Results of GO cellular component classification demonstrated that the UL+CRSR group directly reversed the abnormal metabolism of UL process, especially attenuating the extracellular matrix. Results of GO molecular function classification showed that diverse binding functions and various metabolic and immunological activities were disturbed in UL rats, while CRSR recovered some important disorders of binding functions, and immunological and metabolic activities (28–30). Collectively, the molecular mechanisms of CRSR against the UL process are focused on the arrestment of cell proliferation and ECM-formation genes, intensively tuning a wide range of metabolism disorders.

Results of signaling pathways analysis showed that CRSR can delicately and extensively tune the UL processes. The ECM in UL animals is not only excessive, but also has alterations in its composition (31). The disordered ECM represents the major sign of UL patients (27,31). Altered ECM and its regulating signaling in UL are novel targets for the development of pharmaceutical agents (28,32). Also, excessive ECM directly disturbs extra- and inter-cellular signaling networks (1,2,4,5,33–35). Our results demonstrated four characteristics of CRSR against UL: a) delicately regulated crucial signaling pathways of the UL process, such as PPAR, MAPK, TGF-beta/Smad, and Notch signaling pathways; b) extensively regulated the signaling pathways related to diverse metabolisms, especially xenobiotics biodegradation and metabolism, metabolism of cofactors and vitamins, nucleotide and amino acids metabolism; c) directly suppressed the ECM metabolism; and d) globally impacted the UL process by tuning the endocrine-immune-nervous system.

Our findings shed light on the effective treatment of UL, and provided new insights into the mechanisms underlying the effects of CRSR against the UL process.

## Supplementary material

Click here to view [pdf].
